# Monitoring levodopa oxidation and reduction reactions using surface plasmon resonance on a nanohole array electrode

**DOI:** 10.1186/s11671-023-03930-5

**Published:** 2023-11-28

**Authors:** Hao-Fang Peng, Chih-Kang Chang, Rohit Gupta, Jian-Jang Huang

**Affiliations:** 1 Graduate Institute of Photonics and Optoelectronics, National Taiwan University, Taipei, 10617 Taiwan; 2 Department of Electrical Engineering, National Taiwan University, Taipei, 10617 Taiwan

**Keywords:** Surface plasmon resonance, Nanoholes array, Biosensor, Cyclic voltammetry, L-Dopa

## Abstract

The traditional method of monitoring the oxidation and reduction of biomedical materials usually relies on electrochemical (EC) measurement techniques. Here, we demonstrate a surface plasmon resonance (SPR) method to monitor the oxidation process. Using levodopa L-dopa as the target analyte, a nanohole sensing plate is embedded in the EC electrode to enhance the oxidation signal and generate SPR. Cyclic voltammetry (CV) measurement was first conducted to understand the baseline of EC response of L-Dopa. Then, the redox reactions were simultaneously monitored through SPR measurements during the CV voltage scan. The results showed that the limit of detection using traditional CV reached 1.47 μM while using EC-SPR, the limit of detection improved to 1.23 μM. Most importantly, we found a strong correlation between CV current profiles and the SPR reflection spectra. Our results facilitate detecting electrochemical reactions using an optical probing method.

## Introduction

Cyclic voltammetry (CV) is one of the electrochemical (EC) measurement techniques to study the redox properties in electroanalytical chemistry [1–3]. It involves applying a voltage from low to high and then back to low and analyzing the current response of the target analyte. As the voltage changes, redox reactions occur at the electrode surface, leading to the generation or consumption of electrons, which are detected as changes in current [4]. The resulting electrochemical profile reveals characteristic peaks corresponding to the analyte's oxidation and reduction processes. CV can be used to monitor the status of redox reactions and extract the concentrations. The similarity between EC and biological processes makes CV one of the most versatile methods for biosensing. It is now widely employed to analyze the kinetic, analytical, thermodynamic, and EC behaviors of various biomedical materials [5, 6] and to probe diseases such as diabetes mellitus [7, 8] and glycogen storage disease [9].

Aside from detection using the EC method, surface plasmon resonance (SPR), an optical approach, is a non-intrusive sensing method with extremely high sensitivity. SPR biosensing can be achieved through various types of setups, such as grating [10], Otto [11], and Kretschmann [12] configurations. It is highly sensitive to the dielectric constant of metal and medium at the interface so that it can be used for biomedical sensing, refractive index sensing, and pH sensing [13–15]. EC-SPR combines the advantages of EC and SPR sensing, which typically involves using light incident on a prism and measuring the intensity of reflection at different angles [16, 17]. When SPR occurs, an absorption peak is generated at a specific angle. Moreover, when there is an EC reaction, the angle of the absorption peak shifts. Changes in biomaterials on the metal surface can be monitored from the angle shift [18, 19].

We previously demonstrated a nanohole-array SPR sensor integrated with AC electrokinetic (ACEK) electrodes to characterize the sensing properties of C-reactive protein (CRP) [20]. SPR peaks were found to be dependent on the ACEK voltage and frequency. The results implied that optical signals could represent the status of redox reactions (which are voltage-dependent). In this work, we propose an SPR method to monitor the oxidation process during CV voltage scan, using Levodopa (L-dopa) as the target analyte. L-dopa is a precursor of dopamine. It can cross the blood–brain barrier to increase dopamine neurotransmission and has been considered an important treatment for Parkinson's disease for over half a century [21]. Among various methods [22–27], EC measurement studies the electron transfer process of the L-dopa and is widely employed for clinical detection [28].

In this work, a nanohole sensing plate is embedded in the EC-SPR sensor to enhance the oxidation signal and generate SPR. CV measurement was first conducted to understand the baseline of EC response of L-Dopa. The redox reactions were then simultaneously monitored through the CV and SPR measurements. The limit of detection (LOD) is compared between CV and SPR methods. We found a strong correlation between CV current profiles and the SPR intensity variations.

## Fabrication and measurement

### Nanohole sensor structure

**Fig. 1:**
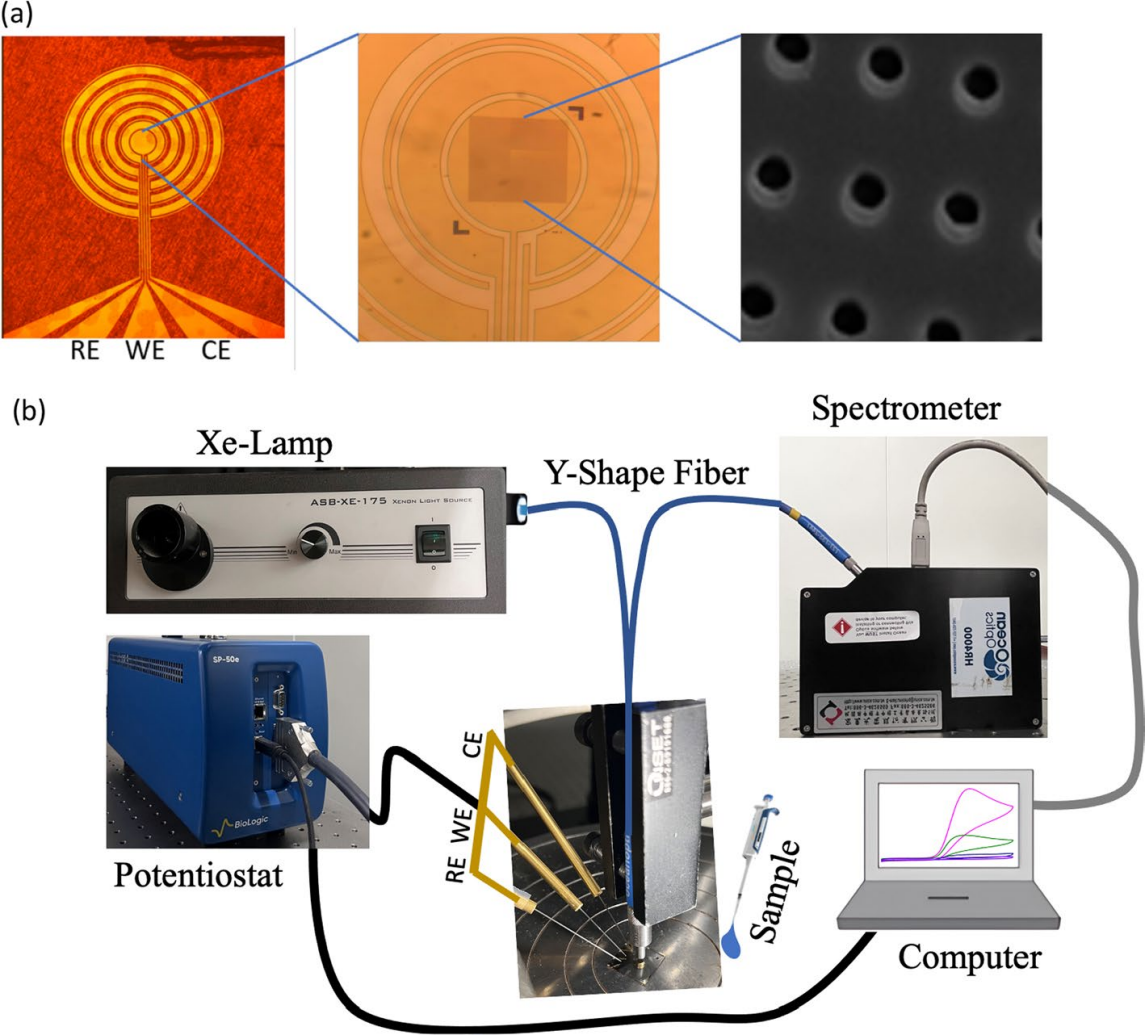
**a** Schematic diagram of the nanohole sensor, **b** measurement setup for EC-SPR experiment.

Similar to the typical alternating-current-electrokinetics (ACEK) biosensor, three electrodes, reference electrode (RE), working electrode (WE), and counter electrode (CE), with the metal stack of Ti/Au (10/150 nm), were deposited on the glass substrate (see Fig. 1a). The concentric rings encircling WE are the electrokinetic flow generator, adeptly collecting target analyte. The Nanohole array, defined by electron-beam lithography, is distributed in the center of WE so that SPR can be effectively generated. The diameter of the nanohole is 250 nm with a period of 750 nm.

### Chemical and materials

3,4-Dihydroxy-L-phenylalanine (levodopa, L-dopa) was purchased from Sigma-Aldrich. It was diluted in a 0.1 M phosphate buffer saline (PBS) solution (pH = 7.4).

### Measurement setup

The measurement setup is shown in Fig. 1b. White light was generated from a Xenon lamp (ASB-Xe-175). It was coupled into the fiber and vertically incident on the nanohole array of the sensor. The reflection light collected by the fiber was analyzed by the spectrometer (Ocean Optics, HR 4000). The potentiostat (SP-50) is employed for cyclic voltammetry measurement. The measurement was conducted by supplying the voltage from the potentiostat to the nanohole sensor. We recorded the optical reflection spectrum from the spectrometer and the electrical current response from the potentiostat simultaneously after the voltage was applied.

### Theory

**Fig. 2:**
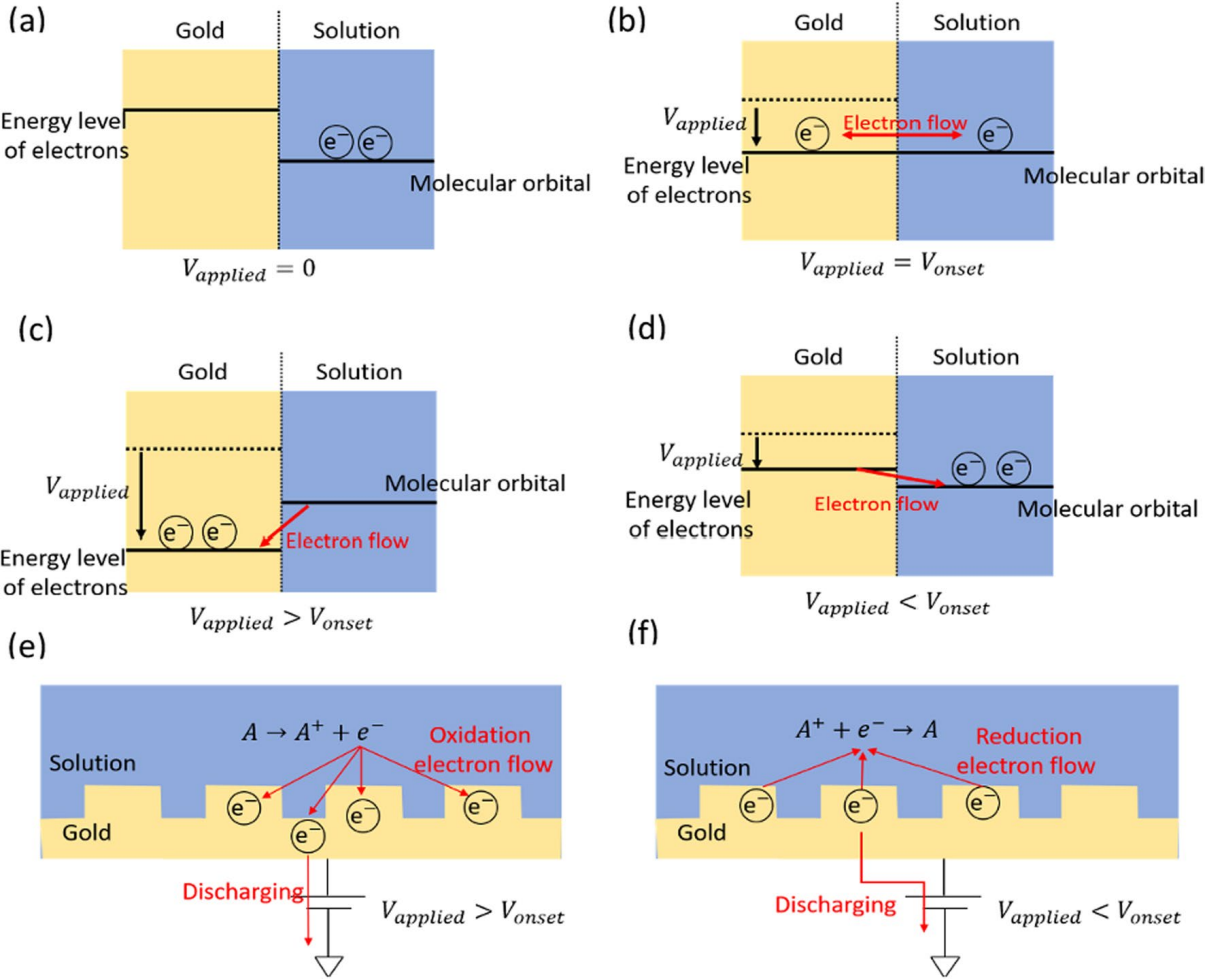
Mechanism of SPR absorptance variations with the EC voltage swing. **a** ${V}_{applied}=0 :$ There is no redox reaction and electron flow. **b**${V}_{applied}={V}_{onset}$: oxidation reaction occurs. **c**${V}_{applied}>{V}_{onset}$: An oxidation current is generated. Electrons flow from the solution to the gold surface. **d** ${V}_{applied}<{V}_{onset}$ (the applied voltage falls back below the onset voltage): A reduction current is generated. The electrons flow from the gold surface to the solution. **e** On the sample surface, electrons flow into the gold when oxidation occurs. **f** When the reduction occurs, electrons move away from the gold surface

We first analyze the SPR-spectral variation during the redox reaction based on the electron energy levels. The electron flow from the analyte to the gold surface is correlated to the SPR spectrum. Considering a gold-solution interface (Fig. 2a) with zero applied voltage (${\mathrm{V}}_{\mathrm{applied}}=0$)), though the electron energy level in the gold layer is higher than that (molecular orbital) in solution, there is no electron flow because the molecular orbitals of the analyte are occupied. When the applied voltage increases to the onset voltage (${V}_{applied}={V}_{onset}$) (see Fig. 2b), the analyte starts to oxidize. The oxidation current will be generated once the applied voltage is larger than the onset voltage (${V}_{applied}>{V}_{onset}$) (see Fig. 2c) because electrons will flow from the occupied molecular orbitals of the analyte to the gold surface. On the other hand, when the applied voltage decreases back to a value below the oxidation voltage, the current is reduced because of the counter electron flow from the gold surface to the vacant molecular orbitals of the analyte, as shown in Fig. 2d.

The EC voltage swing discussed above is employed for CV measurement in a typical EC biosensor. The charging and discharging processes during the voltage swing also change the optical properties of the sensor. In Fig. 2e, when the applied voltage exceeds the onset voltage, electrons flow into the gold surface, causing an increase in local charge density. As a result, the refractive index and dielectric constant become higher [29], leading to an overall increase in absorptance [30]. On the other hand, as shown in Fig. 2f, the discharge of carriers in the gold surface when the applied voltage decreases below the onset leads to a lower absorptance. In this work, the onset voltage during the CV measurement will be correlated to the SPR spectrum so that the dependence of optical absorptance with the redox reaction will be employed for detecting L-dopa of various concentrations.

## Results and discussion

### CV measurement of L-dopa

**Fig. 3:**
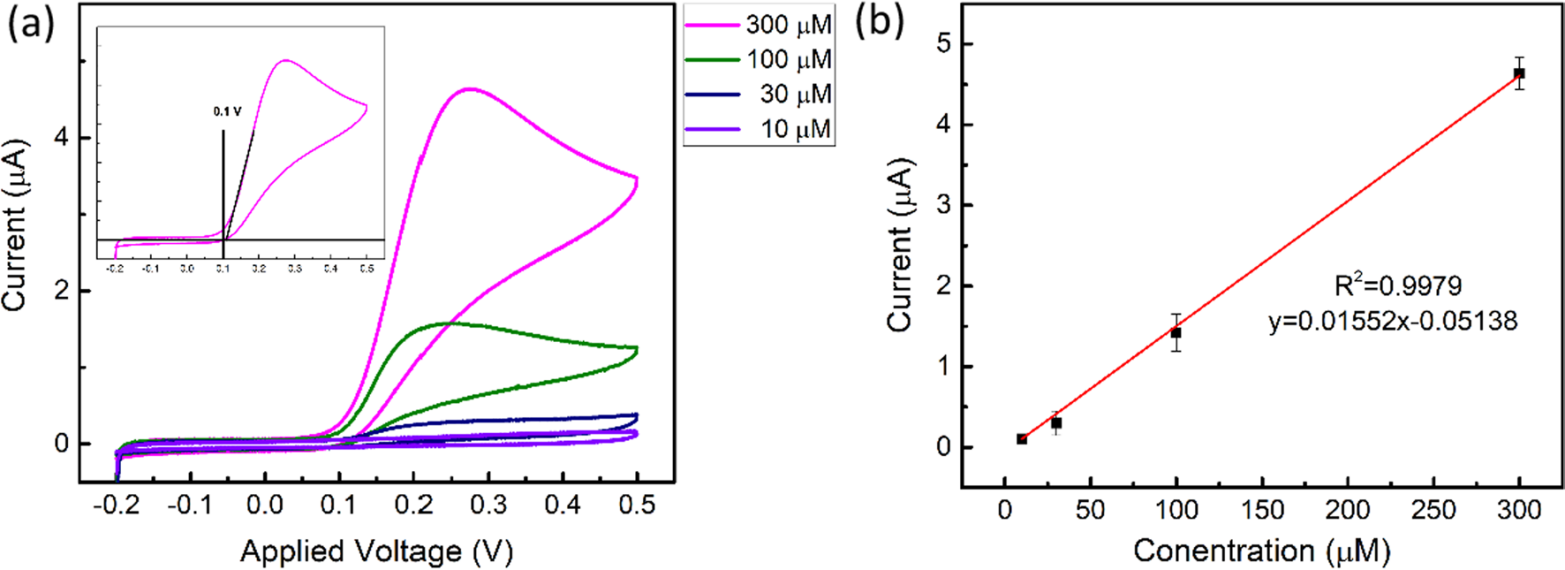
Detecting of L-dopa using CV measurement. **a** Current responses of L-dopa with different concentrations. (Inlet) CV measurement of 300 μM of L-dopa. The onset voltage is 0.1 V using the tangent line method. **b** The corresponding calibration curve of maximum oxidation current extracted from (**a**)

The EC properties of L-dopa are first analyzed. The experiments were performed with a scan rate of 50 mV/sec and voltage scan from -0.2 to 0.5 V. Figure 3a shows the CV characteristics of various L-dopa concentrations in PBS solution (pH = 7.4). The irreversible redox reaction in the CV profiles is mainly attributed to cyclization reaction of the L-dopa oxidation product, levodopa-quinone, at the PBS pH of 7.4 [31, 32]. Generally, the reverse (cathodic) peak is less obvious at a lower scan rate and in a higher pH buffer solution [31]. The magnitude of the current depends on the number of oxidized L-dopa molecules and is correlated to the concentration. Taking the L-dopa with 300 μM in concentration for a close look (see the inlet of Fig. 3a), when the applied voltage approaches ${V}_{onset}$ (around 0.1 V in the inlet), the L-dopa molecules start to oxidize and release electrons, thus rapidly raising the current. As the voltage increases, all the L-dopa on the surface of the metal electrode undergoes oxidation, causing the current to saturate. The magnitude of the current is limited by the rate at which L-dopa diffuses from the upper layer of the solution. On the other hand, in the reverse scan, where the voltage decreases from 0.5 V, the oxidation rate, and thus the current, decreases.

The current–voltage (I-V) relationship near the oxidation onset voltage can be expressed in terms of ${V}_{onset}$ and ${V}_{applied}$ [33]:

1 $$I={I}_{0}\mathrm{exp}\left(\frac{anFe}{RT}({V}_{applied}-{V}_{onset})\right)$$

where $a$ is the transfer coefficient, $n$ is the stoichiometric number of electrons involved in the EC reaction, $F$ is the Faraday constant, $e$ is the elementary charge, $R$ is the gas constant, $T$ is the Kelvin temperature. For L-dopa with a concentration of 300 μM, ${V}_{onset}$ is determined by extrapolating the I-V curve in the linear region (0.14 ~ 0.2 V) of the Fig. 3a inlet. ${V}_{onset}$ of 0.1 V is obtained. In the following sections, we will use this value to determine the onset of the oxidation reaction.

Figure 3b shows the calibration curve by extracting the maximum current value of each concentration in Fig. 3a. 5 devices of each concentration were characterized. The We adopt the following equation to estimate the limit of detection (LOD) [34]:

2 $$\mathrm{LOD}=3\times \frac{\mathrm{SD}}{\mathrm{S}}$$

where SD is the y-axis-intercept standard deviation of response, and S is the slope of the calibration curve.

The SD, extracted from Fig. 3b, is 0.0076. Based on Eq. (2), our device's LOD using the CV method is 1.47 μM. Among similar CV methods to detect L-dopa on the un-modified electrodes [35–37], our approach demonstrates good LOD, mainly because of the nanoholes' larger chemical reaction surface area.

### SPR spectral change during the redox reaction

In this section, we measure the in-situ SPR spectrum during the CV measurement. The reflection spectra measured at time t are normalized to the initial spectra by

3 $$\Delta \mathrm{R}(\mathrm{t})=\frac{\mathrm{R}\left(\mathrm{t}\right)-\mathrm{R}(0)}{\mathrm{R}(0)}$$

where R(0) represents the initial spectrum at t = 0, the initial spectrum at the start of measurement, R(t) is the spectrum measured at time t, and t indicates the time after applying the cyclic voltammetry.

**Fig. 4:**
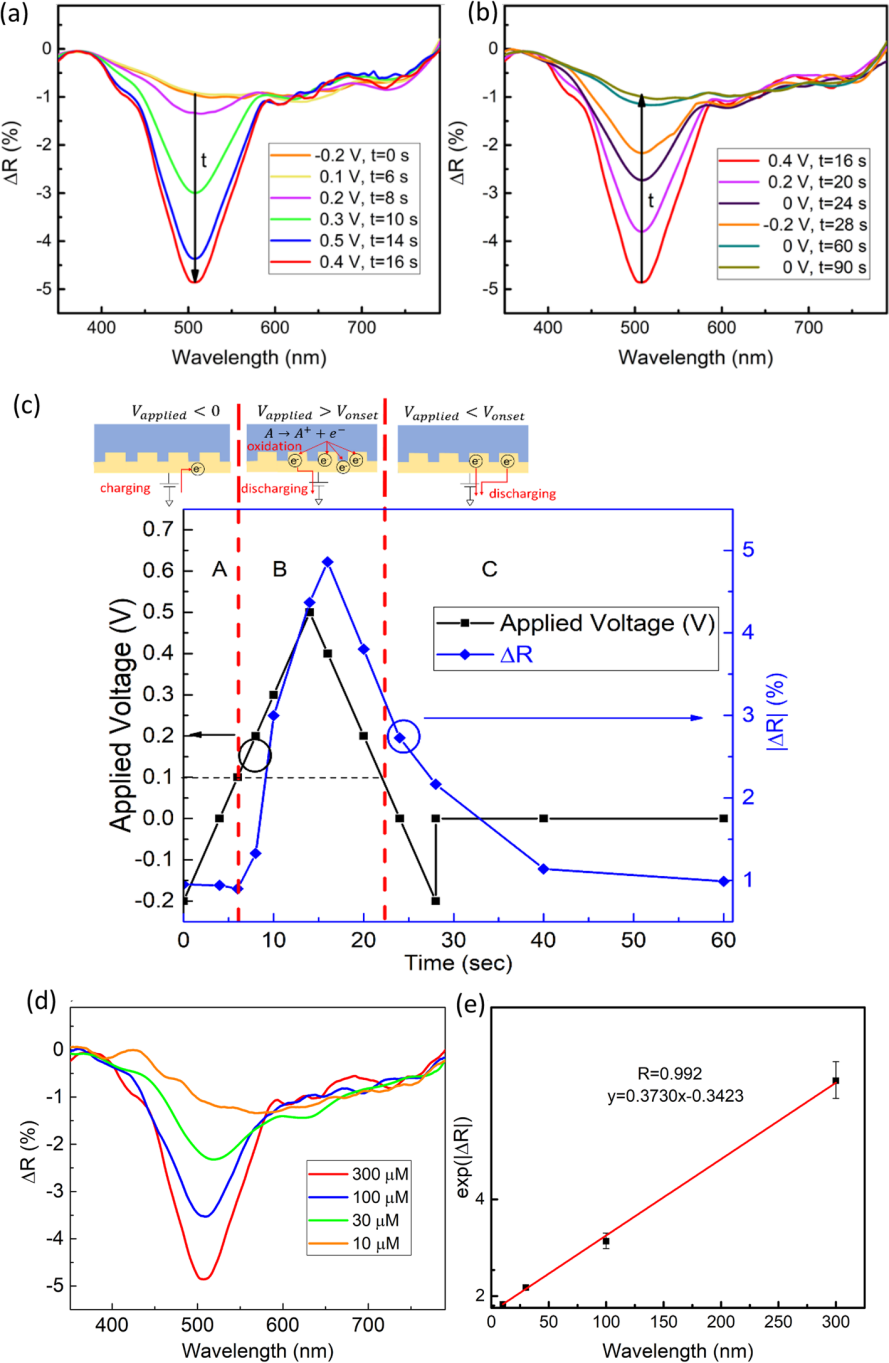
The measurement result of the SPR experiment. **a** ∆R spectral changes with time. When oxidation occurs, electrons flow into metal, and ∆R increases. **b** The rate of electron dissipation from the metal surface is greater than the rate of electron generation by oxidation, leading to a decrease of ∆R. **c** Transient correlation between peak ∆R and applied voltage. The corresponding charge movement is schematically drawn on top of the figure. **d** Spectra at the maximum ∆R of various L-dopa concentrations. The maximum ∆R of all concentrations occurs at the voltage of 0.4 V. **e** Corresponding calibration curve of peak intensity at the maximum ∆R of various concentrations

Taking 300 μM L-dopa in PBS solution (pH = 7.4) as an example, the SPR responses are represented by the peak optical reflection changes. As shown in Fig. 4a, the peak value of ∆R increases in the negative direction with the applied voltage. The ∆R change is mainly attributed to the increased localized electron density and the corresponding dielectric constant and optical absorption increase. On the other hand, as shown in Fig. 4b, when we bring the voltage down from 0.4 V, ∆R decreases because carriers move away from the nanohole surface.

The correlation between the peak optical reflection variation and the scanning voltage is plotted in the timeline to monitor the progress of the reaction. In Fig. 4c, we divide the reaction into several regions based on the onset voltage. In region A, the applied voltage increment from negative (toward positive) results in a slight increase in electron concentration. The reflectivity corresponds to the energy level in Fig. 2a, at which no oxidation current appears. When the applied voltage increases beyond the onset voltage (0.1 V), the reaction enters region B, where the oxidation reaction starts (see the energy level diagram in Fig. 2b). Once oxidation occurs, the electron concentration on the metal surface increases rapidly, leading to a significant increase in light absorptance. The energy level diagram in Fig. 2c illustrates the phenomenon. In region B, the peak voltage of 0.5 V is applied at t = 14 s. Because of the time required for electron movement, the maximum ∆R occurs at the following data point of extraction, i.e., t = 16 s. Furthermore, in region C, the rate of electron dissipation from the metal surface becomes larger than the electron generation rate from oxidation. We observe a decrease in electron concentration and |∆R|.

### SPR spectra of various analyte concentrations during the redox reaction

The change in ∆R depends on the electron concentration on the metal surface, which is determined by the number of electrons flowing through the metal surface during the redox process. In other words, ∆R is dependent on the concentration of the analyte.

The experiments of monitoring L-dopa of different concentrations were performed under the following conditions: the scan rate is 50 mV/sec, the initial voltage is -0.2 V, and the maximum voltage is 0.5 V. ∆R(t) of different L-dopa concentrations was recorded. Then, the ∆R spectra with the maximum reflectivity change during the voltage scan are shown in Fig. 4d for L-dopa with different concentrations. The calibration curve in Fig. 4e is plotted by extracting each concentration's peak ∆R in Fig. 4d. 5 devices of each concentration were characterized. From Eq. (2), the LOD obtained using the SPR method is 1.23 μM. Compared to our CV measurement on the nanohole sensor, the EC-SPR method has a slightly lower LOD. The similar level of LOD between CV and EC-SPR measurements implies that system noise may be a limiting factor instead of the measurement method.

## Discussions of the EC-SPR monitoring on the L-dopa redox reaction

In contrast to traditional EC-SPR measurements, which monitor peak wavelength shift or peak angular shift from the change of solution’s dielectric constant [18], our experiment focuses on the correlation of the oxidation process with the SPR reflection spectra. From the application point of view, because we extract ∆R(t) at only the peak wavelength, the light source used for the proposed sensor can be fixed to a specific wavelength (506 nm in our results) without scanning through the whole spectrum. In addition, our nanohole sensor is highly sensitive to the oxidation current that flows into the metal surface, making it suitable for redox reaction sensing.

During the measurement, the L-dopa molecules inevitably undergo adsorption on the nanohole- EC electrodes, even though the device was washed by PBS/DI water/Acetone/IPA (Isopropanol Alcohol)/DI water between run to run. For L-dopa concentration of 300 μM, CV peak current variation can be maintained within 3% for measurements on the same sensor up to 10 times. The variation will gradually become larger for more measurements. The variation also increases with the concentration as surface adsorption is more severe. For a larger concentration range (between 10 μM and 5 mM), we observe nonlinearity in the calibration curves for both CV and SPR measurements. As a result, in this work, we focus on L-dopa concentrations up to only 300 μM.

For Parkinson's disease patients undergoing L-dopa therapy, monitoring the concentration of L-dopa in biological fluids such as urine and blood is paramount. Studies have shown that the concentration of L-dopa in urine ranges from approximately 18.9 to 237.3 μM [38] and around 14.7 μM in the bloodstream [39]. Thus, the range of concentration in this work is suitable for practical applications. The LOD achieved by our method (1.23 μM) is sufficient for detecting L-dopa in the blood and urine of Parkinson's disease patients. The unique oxidation onset voltage of 0.1 V can also be used to identify L-dopa molecules. For applications other than detecting L-dopa, the oxidation onset voltage can differentiate the components of drugs because different drugs exhibit distinct onset voltages. Multiple current peaks can be observed during voltage scanning, and our method can be applied in this context.

## Conclusion

In this work, we analyze the correlation of redox reaction and SPR spectra based on the mechanism of electron flow in the energy levels. The CV properties of the L-dopa in PBS were first characterized as the baseline. On the un-modified electrode, we achieve a low LOD of 1.47 μM using the CV method, which is attributed to nanoholes in the metal sensing pad. As for EC-SPR sensing, we found a strong correlation between the oxidation current and the SPR reflection spectral changes. It confirms the idea of monitoring redox reactions using SPR spectra. The proposed method can satisfy the need to facilitate electrochemical detection by using an optical probing method. It also provides a parallel approach to analyzing biomaterials and the CV measurement.

## Data Availability

Data underlying the results presented in this paper are not publicly available at this time but may be obtained from the authors upon reasonable request.

## References

[1] Noémie E (2018). A practical beginner’s guide to cyclic voltammetry. J Chem Educ.

[2] Pumidech P, Venton BJ (2020). Recent advances in fast-scan cyclic voltammetry. Analyst.

[3] Heinze J, Doz P (1984). Cyclic voltammetry-“electrochemical spectroscopy” new analytical methods. Angew Chem Int Ed.

[4] Laviron E, Roullier L (1980). General expression of the linear potential sweep voltammogram for a surface redox reaction with interactions between the adsorbed molecules: applications to modified electrodes. J Electroanal Chem Interfacial Electrochem.

[5] Dăscălescu D, Apetrei C (2021). Voltammetric determination of levodopa using mesoporous carbon-modified screen-printed carbonsensors. Sensors.

[6] Gulaboski R, Kokoskarova P, Petkovska S (2020). Analysis of drug-drug interactions with cyclic voltammetry: an overview of relevant theoretical models and recent experimental achievements. Anal Bioanal Electrochem.

[7] Chevion S (1997). The antioxidant properties of thioctic acid: characterization by cyclic voltammetry. Biochem Mol Biol Int.

[8] Chevion S, Chevion M (2000). Antioxidant status and human health. Use of cyclic voltammetry for the evaluation of the antioxidant capacity of plasma and of edible plants. Ann NY Acad Sci.

[9] Koren E (2009). Total oxidant-scavenging capacities of plasma from glycogen storage disease type Ia patients as measured by cyclic voltammetry, FRAP and luminescence techniques. J Inherit Metab Dis.

[10] Ebbesen TW (1998). Extraordinary optical transmission through sub-wavelength hole arrays. Nature.

[11] Lee Y, et al. Otto configuration based surface plasmon resonance with tunable air-gap using piezoactuator. In: International Conference on Optical MEMS and Nanophotonics (OMN). 2019. pp. 64–65.

[12] Menon P S, et al. Kretschmann based surface plasmon resonance for sensing in visible region. In: IEEE 9th International Nanoelectronics Conferences (INEC). 2019. pp. 1–6. 10.1109/INEC.2019.8853847.

[13] Kim HM (2009). Detection of biomolecular binding through enhancement of localized surface plasmon resonance (LSPR) by gold nanoparticles. Sensors (Basel).

[14] Shuai W (2018). The investigation of an LSPR refractive index sensor based on periodic gold nanorings array. J Phys D Appl Phys.

[15] Hao J (2009). Near-infrared optical response of thin film pH-sensitive hydrogel coated on a gold nanocrescent array. Opt Express.

[16] Geilfuss D (2022). Can classical surface plasmon resonance advance via the coupling to other analytical approaches. Front Anal Sci.

[17] Li C (2022). Novel electrochemical-surface plasmon resonance (EC-SPR) sensor for amphetamine-type stimulants detection based on molecularly imprinted strategy. Sens Actuators B Chem.

[18] Ribeiro A (2022). Electrochemistry combined-surface plasmon resonance biosensors: a review. Trends Anal Chem.

[19] Mayer KM, Hafner JH (2011). Localized surface plasmon resonance sensors. Chem Rev.

[20] Ma SC (2023). Voltage-modulated surface plasmon resonance biosensors integrated with gold nanohole arrays. Biomed Opt Express.

[21] Abbott A (2010). Levodopa: the story so far. Nature.

[22] Elbarbry F (2019). A new validated HPLC method for the determination of levodopa: application to study the impact of ketogenic diet on the pharmacokinetics of levodopa in Parkinson's participants. Biomed Chromatogr.

[23] Hormozi-Nezhad MR (2017). Simple and rapid detection of L-dopa based on in situ formation of polylevodopa nanoparticles. Sens Actuators B Chem.

[24] Xiang G (2022). A sensitive photoelectrochemical sensor for levodopa detection using benzothiadiazole-based conjugated microporous polymer-coated graphene heterostructures. ACS Appl Mater Interfaces.

[25] Brunetti B (2014). A disposable electrochemical biosensor for L-DOPA determination in undiluted human serum. Electrochem Commun.

[26] Ensafi AA (2010). Sequential determination of benserazide and levodopa by voltammetric method using chloranil as a mediator. J Braz Chem Soc.

[27] Reddaiah K (2013). Poly(Xylene Cyanol FF) chemical sensor for the boost up of electro-catalytic oxidation of L-Dopa in the presence of ascorbic acid and uric acid: a voltammetric study. Sens Lett.

[28] Crapnell RD, Banks CE (2023). Electroanalytical overview: the determination of levodopa (L-DOPA). ACS Meas Sci Au.

[29] Feynman RP (1965). The Feynman lectures on physics.

[30] Hoener BS (2017). Spectral response of plasmonic gold nanoparticles to capacitive charging: morphology effects. J Phys Chem Lett.

[31] Shahrokhian S, Asadian E (2009). Electrochemical determination of l-dopa in the presence of ascorbic acid on the surface of the glassy carbon electrode modified by a bilayer of multi-walled carbon nanotube and poly-pyrrole doped with tiron. J Electroanal Chem.

[32] Wang Y (2021). A strategy to differentiate dopamine and levodopa based on their cyclization reaction regulated by pH. Anal Chim Acta.

[33] Mabbott GA (1983). An introduction to cyclic voltammetry. J Chem Educ.

[34] Shrivastava A (2011). Methods for the determination of limit of detection and limit of quantitation of the analytical methods. Chron Young Sci.

[35] Tai LC (2019). Wearable sweat band for noninvasive levodopa monitoring. Nano Lett.

[36] Bergamini MF (2005). A disposable EC sensor for the rapid determination of levodopa. J Pharm Biomed Anal.

[37] Li LJ (2008). Determination of levodopa in pharmaceutical preparations by irreversible biamperometry. Chin Chem Lett.

[38] Baranowska I, Płonka J (2008). Determination of levodopa and biogenic amines in urine samples using high-performance liquid chromatography. J Chromatogr Sci.

[39] Nyholm D (2013). Pharmacokinetics of levodopa, carbidopa, and 3-O-methyldopa following 16-hour jejunal infusion of levodopa-carbidopa intestinal gel in advanced Parkinson's disease patients. AAPS J.

